# A Lunatic's Relatives

**Published:** 1894-07-28

**Authors:** 


### A LUNATIC'S RELATIVES.

AMONG the troubles and anxieties of an asylum doctor's life the friends of his patients may certainly be reckoned. They are of all varieties, but a considerable number are more than kin, in that they also are not wholly free from the taint of madness, and often decidedly less than kind. A husband who brought his wife to the Morningside Asylum, Edinburgh, was obliging enough to give a hint as to the proper mode of treating the case. He had been in the habit of thrashing her twice a week, and thought the discipline should be continued. We could wish this poor woman no worse fate than that she should be cured and forced to return to her amiable spouse. The super-sensitive relatives—the opposite of this kind husband—are, however, almost as great a nuisance. They come to the asylum weeping and trembling, bewailing to the lunatic himself his sad condition, till they upset his composure, and cause no little extra trouble to attendants who are sufficiently hard worked already. Still even these are less undesirable visitants than the drunken father who, as we are informed by Dr. Elkins, of the Edinburgh Asylum, finding his insane daughter uninterested in his conversation promptly gave her a black eye. One would incline to say, in face of such instances, that a lunatic is better without visitors; but this is not so. Very few patients completely lose their own identity, a considerable number know not only who they are, but where they are, and the knowledge is a grief and a humiliation to them. These feelings are aggravated by the notion that those who once loved them, who were once their companions have deserted them. And often enough they seem to be forgotten. Their kinsfolk bring them to the asylum, and then, glad to be rid of the incubus, never return to ask how they fare. Oftenest is this the case, sad to say, with husbands and wives, the tie of wedlock, close and permanent as it is, seems to snap at the asylum gates; nor are children too devoted to feeble-minded parents; while mothers and maiden aunts seem to be the most loyal to their insane relatives. Doubtless the visits of these devoted women are not an unmixed benefit; they are liable to unreasonable emotion; they put leading questions to the patients to make them complain, and accept their answers as undoubted truth. But at their worst they do less harm than those who make the asylum as complete a severance as the grave.

